# Functional Characterization of the Ciliate *Stylonychia lemnae* Serotonin *N*-Acetyltransferase, a Pivotal Enzyme in Melatonin Biosynthesis and Its Overexpression Leads to Peroxidizing Herbicide Tolerance in Rice

**DOI:** 10.3390/antiox13101177

**Published:** 2024-09-27

**Authors:** Kyungjin Lee, Kyoungwhan Back

**Affiliations:** Department of Molecular Biotechnology, College of Agriculture and Life Sciences, Chonnam National University, Gwangju 61186, Republic of Korea; nicekj7@hanmail.net

**Keywords:** archaea, ciliophoran, melatonin, *Osl20*, serotonin *N*-acetyltransferase, *Stylonichia lemnae*, transgenic rice

## Abstract

Serotonin *N*-acetyltransferase (SNAT) is a pivotal enzyme for melatonin biosynthesis in all living organisms. It catalyzes the conversion of serotonin to *N*-acetylserotonin (NAS) or 5-methoxytrypytamine (5-MT) to melatonin. In contrast to animal- and plant-specific *SNAT* genes, a novel clade of archaeal *SNAT* genes has recently been reported. In this study, we identified homologues of archaeal *SNAT* genes in ciliates and dinoflagellates, but no animal- or plant-specific *SNAT* homologues. Archaeal *SNAT* homologue from the ciliate *Stylonychia lemnae* was annotated as a putative *N*-acetyltransferase. To determine whether the putative *S. lemnae SNAT* (*SlSNAT*) exhibits SNAT enzyme activity, we chemically synthesized and expressed the full-length *SlSNAT* coding sequence (CDS) in *Escherichia coli*, from which the recombinant SlSNAT protein was purified by Ni^2+^ affinity column chromatography. The recombinant SlSNAT exhibited SNAT enzyme activity toward serotonin (*K*_m_ = 776 µM) and 5-MT (*K*_m_ = 246 µM) as substrates. Furthermore, *SlSNAT*-overexpressing (SlSNAT-OE) transgenic rice plants showed higher levels of melatonin synthesis than wild-type controls. The SlSNAT-OE rice plants exhibited delayed leaf senescence and tolerance against treatment with the reactive oxygen species (ROS)-inducing herbicide butafenacil by decreasing hydrogen peroxide (H_2_O_2_) and malondialdehyde (MDA) levels, suggesting that melatonin alleviates ROS production in vivo.

## 1. Introduction

Organisms from all kingdoms of life synthesize melatonin [1]. First discovered in 1958, melatonin was identified as a pineal factor that lightened melanocytes [2,3]. Although its skin-lightening effects were restricted to amphibians, many other biological activities have been documented, among which the best-described roles are in the regulation of the sleep–wake cycle and cellular redox homeostasis in animals [4,5]. Additionally, melatonin plays key roles in survival by orchestrating protein quality control, such as the chaperone network, autophagy, and the ubiquitin–proteasome system, in both animals and plants [6,7,8]; it also acts as a potent antioxidant by scavenging a range of harmful oxidants [9].

Melatonin is biosynthesized through a four-step sequential enzymatic reaction with tryptophan as the first substrate in all organisms [10,11]. The common last intermediate is serotonin, produced by two enzymes—tryptophan hydroxylase (TPH) and aromatic amino acid decarboxylase (AADC) in animals, and tryptophan decarboxylase (TDC) and tryptamine 5-hydroxylase (T5H) in plants. As for the last two enzymatic reactions, arylalkylamine *N*-acetyltransferase (AANAT) (also designated as SNAT) and *N*-acetylserotonin *O*-methyltransferase (ASMT) are involved in melatonin biosynthesis. These enzymes catalyze the conversion of serotonin to either *N*-acetylserotonin (NAS) or 5-methoxytryptamine (5-MT) in both animals and plants. NAS is synthesized by SNAT, whereas 5-MT is synthesized by ASMT, followed by melatonin synthesis through the action of SNAT. NAS leads to melatonin synthesis by ASMT. Interestingly, NAS is converted back to serotonin by the action of NAS deacetylase in plants, resulting in the accumulation of serotonin (rather than melatonin) [12]. Therefore, the pathway from serotonin → 5-MT → melatonin can overcome the reverse reaction of classical melatonin biosynthesis (serotonin → NAS → melatonin), which leads to enhanced melatonin production, as observed in plants exposed to various stressors [13].

Among the four enzymes, SNAT is thought to play pivotal roles because it is closely associated with both rhythmic melatonin synthesis in animals [14] and the physiological functions of melatonin in plants [15]. Correspondingly, a range of *SNAT* genes have been cloned from numerous animal and plant species, although there is no apparent amino acid sequence identity between animal and plant *SNAT* genes [11]. *SNAT* exists as a single copy in animals, whereas plants harbor at least two copies [16]. Surprisingly, a substantial portion of NAS synthesis occurs in an *AANAT*-independent manner in hamsters and rats, suggesting the existence of an alternative SNAT enzyme, such as protein *N*-acetyltransferase (NAT) [17]. Consistent with the predictions of Slominski [17], it was recently reported that human Naa50, a NAT family protein, exhibited SNAT enzyme activity [18]. Human Naa50 can catalyze the conversion of both serotonin and 5-MT into NAS and melatonin, similar to animal and plant SNAT proteins [16,19]. The successful cloning of an alternative *SNAT* from humans, *Naa50*, was attributed to the cloning of archaeon *SNAT* because they are functional orthologues [18,20]. Unlike the animal-specific *AANAT* and plant-specific *SNAT* genes, human *Naa50* or archaeal *SNAT* orthologues are distributed in all kingdoms of life, including ciliates and dinoflagellates, because melatonin is ubiquitously present throughout nature. For example, the ciliate *Tetrahymena pyriformis* and dinoflagellate *Gonyaulax polyedra* are representative species that synthesize melatonin, but neither *AANAT* nor *SNAT* orthologues have been identified in their genomes [1].

Here, we found many human *Naa50* orthologues in ciliates and dinoflagellates; the ciliate *Stylonychia lemnae Naa50* orthologue was annotated as a putative NAT (GenBank accession number CDW73552). To determine whether the protein product of the *Naa50* orthologue from *S. lemnae* exhibits SNAT enzyme activity, we expressed the putative *S. lemnae SNAT* (*SlSNAT*) in *Escherichia coli*, from which the recombinant SlSNAT protein was purified and subjected to analysis of SNAT enzyme kinetics in vitro. Furthermore, we performed in vivo functional analysis by transforming the *SlSNAT* gene into rice to determine whether its ectopic overexpression was coupled with melatonin biosynthesis in transgenic plants along with enhanced tolerance to oxidative stress.

## 2. Materials and Methods

### 2.1. Codon-Optimized Chemical Synthesis of S. lemnae SNAT Gene

Based on the amino acid sequence information of *S. lemnae* SNAT (GenBank accession number CDW73552), the full-length nucleotides of *S. lemnae SNAT* with the length of 546 bp were codon optimized by reference of rice *SNAT2* codon and custom synthesized (Bioneer, Daejeon, Republic of Korea).

### 2.2. Escherichia coli Expression, Production, and Recombinant S. lemnae SNAT Protein Purification

Expression, production, and recombinant protein purification of SlSNAT have been described previously elsewhere [18,21]. In brief, the full-length synthetic *SlSNAT* gene was amplified by PCR by using a primer set (*SlSNAT* forward primer, 5′-ACC ATG GCC ATG CCG GCG CCC GAG GCG-3′; *SlSNAT* reverse primer, 5′-CTC GAG CTG CGA CGT GGT CGA CTG-3′) with a template plasmid containing the synthetic *SlSNAT* DNA such as pBHA-SlSNAT which was synthesized by Bioneer. The PCR product was ligated into the TA vector (RBC Bioscience, New Taipei City, Taiwan) followed by plasmid purification of TA-SlSNAT. The TA-SlSNAT plasmid was digested with *Nco*I and *Xho*I restriction enzymes. Then, the *Nco*I and *Xho*I insert of *SlSNAT* DNA was ligated into the same restriction sites of the *E. coli* expression vector pET32b (Novagen, Madison, WI, USA) to construct the pET32b-SlSNAT vector construct. As for pET300-SlSNAT vector construction, the full-length *SlSNAT* DNA of 546 nucleotides in length was amplified by PCR using a primer set (forward primer 5′-AAA AAG CAG GCT CCA TGC CGG CGC CCG AGG-3′; reverse primer 5′-AGA AAG CTG GGT CTA CTG CGA CGT GGT CGA-3′) using the synthetic *SlSNAT* DNA as the template. The resulting PCR product was further amplified by PCR using an *attB* primer set [18]. The full-length *SlSNAT* PCR product was cloned into the pDONR221 gateway vector (Invitrogen, Carlsbad, CA, USA) via the BP recombination reaction. The pDONR221-SlSNAT gene entry vector was then recombined with the pET300 destination vector (Invitrogen) via LR recombination to yield pET300-SlSNAT. Both the pET32b-SlSNAT and pET300-SlSNAT plasmids were transformed into *E. coli* strain BL21(DE3) (Invitrogen, Carlsbad, CA, USA). Further *E. coli* culture, isopropyl-β-D-thiogalactopyranoside (IPTG; Sigma, St. Louis, MO, USA) treatment, and affinity (Ni^2+^) purification were described in detail previously [21]. The purified thioredoxin (Trx)-tagged SlSNAT fusion protein was mixed with the equal volume of glycerol and stored at −80 °C until use. Protein concentrations were determined using the Bradford method and a protein assay dye (Bio-Rad, Hercules, CA, USA).

### 2.3. Homology and Phylogenetic Analysis

The analysis of amino acid sequence homology search using human Naa50 as a query was carried out with the BLASTp tool in the non-redundant protein sequences databases at the National Center for Biotechnology Information (http://www.ncbi.nlm.nih.gov/, accessed on 26 August 2019). Phylogenetic tree analysis was achieved by using the BLAST-Explorer program (version 2, Information Genomique & Structurale, Marseille, France) [22].

### 2.4. Enzymatic Assays for SNAT

The enzymatic assay for SNAT was performed in a 100 µL final volume containing 0.8 µg of the purified recombinant Trx-SlSNAT protein, 0.5 mM serotonin (or other substrates), and 0.5 mM acetyl-CoA in 100 mM potassium phosphate (pH 8.8 or varying pH) at 55 °C (or other temperatures) for 30 min. Twenty microliters of enzymatic reaction was analyzed by reverse phase high-performance liquid chromatography (HPLC) as described previously [23]. The Lineweaver–Burk plots were employed to calculate substrate affinity (*K*_m_) and the maximum reaction rate (*V*_max_) using two substrates such as serotonin and 5-methoxytryptamine. The analysis was performed in triplicate.

### 2.5. Generation of Transgenic Rice Plants Overexpressing the Synthetic SlSNAT Gene

In order to deliver the synthetic *SlSNA*T DNA into the rice genome, we utilized a pIPKb002 gateway binary vector. The pIPKb002 binary vector was kindly provided by Dr. J. Kumlehn (Leibniz Institute of Plant Genetics and Crop Plant Search, Gatersleben, Germany) [24]. In brief, the pDONR221-SlSNAT gene entry vector was then recombined with the pIPKb002 destination vector via LR recombination to yield pIPKb002-SlSNAT, which was then transformed into *Agrobacterium tumefaciens* strain LBA4404. *Agrobacterium*-mediated rice transformation using the calli generated from a japonica rice cultivar called Dongjin was employed to generate transgenic rice plants as described previously [25].

### 2.6. Melatonin Measurement from the SlSNAT Overexpression (SlSNAT-OE) Transgenic Rice Plants

Melatonin levels were measured in frozen rice leaf samples (0.1 g) using the TissueLyser II (Qiagen, Tokyo, Japan). Melatonin was quantified by high-performance liquid chromatography (HPLC) with a fluorescence detector system (Waters, Milford, MA, USA) as described previously [18].

### 2.7. Senescence Treatment in the SlSNAT-OE Transgenic Rice Plants

Rice leaves from rice grown in soil for 5 weeks in a glass house at 28 °C under a 12 h light/12 h dark cycle at a photosynthetic photon flux density of 150 µmol m^−2^ s^−1^ were detached for an in vitro senescence experiment. Groups of 10 segments (detached fourth and fifth leaves) were transferred to 50 mL polypropylene conical tubes containing 25 mL of water. The samples were incubated for 12 d under the same growth conditions as described above. The entire rice leaves were frozen in liquid nitrogen and pulverized to a powder using a TissueLyser II instrument (Qiagen). As for the measurement of chlorophyll contents, the powder (100 mg) was extracted with 1 mL of 0.1 M NH_4_OH (containing 80% acetone). Chlorophyll concentrations were determined at wavelengths of 647, 644, and 750 nm using a spectrophotometer (Optizen POP-Bio; Mecasys, Daejeon, Republic of Korea) according to Porra et al. [26]. The levels of malondialdehyde (MDA) were measured at wavelengths of 440, 532, and 600 nm using a spectrophotometer (Optizen POP-Bio) as described previously [27].

### 2.8. Total RNA Isolation and Reverse Transcription–Polymerase Chain Reaction (RT-PCR)

Total RNA was isolated using a Ribospin Plant Kit (GeneAll Biotechnology Co., Seoul, Republic of Korea). RT-PCR was conducted using a rice *ubiquitin-5 gene* (*UBQ5*) as the loading control. The sequences of primers were listed previously [27]. As for the real-time PCR analysis, a Mic qPCR Cycler system (Bio Molecular Systems, Coomera, Queensland, VIC, Australia) with the Luna Universal qPCR Master Mix (New England Biolabs, Ipswich, MA, USA) was utilized. The expression of genes was analyzed using Mic’s RQ software v2.2 (Bio Molecular Systems) and normalized to *UBQ5* as described previously [28].

### 2.9. Tolerance against Peroxidizing Herbicide Butafenacil

Surface-sterilized dehusked rice seeds were sown on half-strength Murashige and Skoog (MS) medium [27]. The 7-day-old seedlings collected from MS medium were incubated in 50 mL polypropylene conical tubes containing 0.1 µM butafenacil (a kind gift from Dr. Guh (Chonnam National University, Gwangju, Republic of Korea)) for 12 h in the dark followed by a 12 h light/12 h dark cycle for 48 h. Cellular leakage in medium was determined using a conductivity meter (Cole-Parmer Instrument LLC, IL, USA) as described previously [29]. Hydrogen peroxide contents were quantified by an OxiTec™ Hydrogen Peroxide/Peroxidase (H_2_O_2_) Assay Kit (Biomax, Guri-si, Republic of Korea).

### 2.10. Statistical Analysis

The data were analyzed by analysis of variance using IBM SPSS Statistics 23 software (IBM Corp. Armonk, NY, USA) as described previously [27].

## 3. Results

### 3.1. Selection and Chemical Synthesis of Stylonychia lemnae SNAT Gene

**Figure 1:**
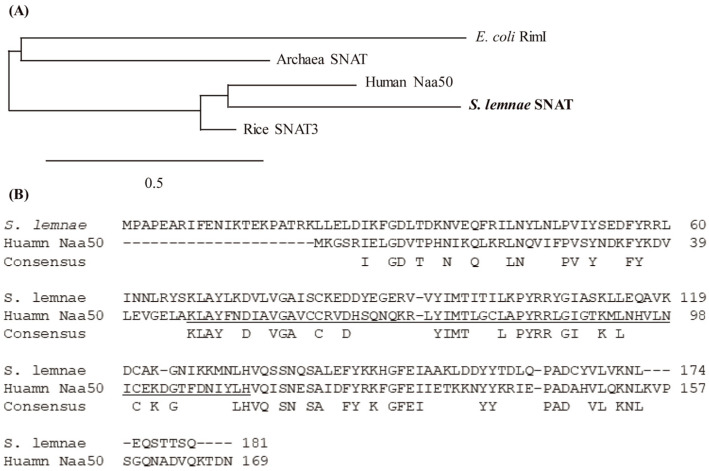
(**A**) Phylogenetic tree of *Stylonichia lemnae SNAT* and archaeal ortholog genes. The scale bar represents 0.4 substitutions per site. *S. lemnae SNAT* is written in bold for emphasis. (**B**) Amino acid sequence identity and similarity between *S. lemnae* SNAT and human Naa50 (SNAT). The conserved acetyl-coenzyme-A-binding sites are underlined. Dashes denote gaps. GenBank accession numbers are archaea SNAT (NC_002689), *E. coli* RimI (WP_137442509), human Naa50 (BAB14397), rice SNAT3 (AK241100), and *S. lemnae* SNAT (CDW73552).

Analysis using BLASTp (http://www.ncbi.nlm.nih.gov/, accessed on 26 August 2019) indicated that human Naa50 harboring SNAT enzyme activity [18] exhibited ~38% identity to a putative SNAT protein of *S. lemnae* consisting of 181 amino acids (aa) with 83% query cover value. Phylogenetic analysis indicated that the putative *S. lemnae* SNAT (SlSNAT) protein was the closest orthologue of human Naa50 belonging to the archaeal SNAT clade (Figure 1A). The two protein sequences were aligned using BLASTp, revealing that SlSNAT had 38% aa identity to human Naa50 (Figure 1B). The putative SlSNAT was annotated as a member of the protein NAT family carrying a region with identity to *E. coli* RimI, ranging in size from 40 to 176 aa. It was recently reported that RimI, an *N*-terminal protein acetyltransferase, also exhibited SNAT enzyme activity [21]. Taken together, the results of these in silico analyses suggested that the putative *SlSNAT* may exhibit SNAT enzyme activity.

**Figure 2:**
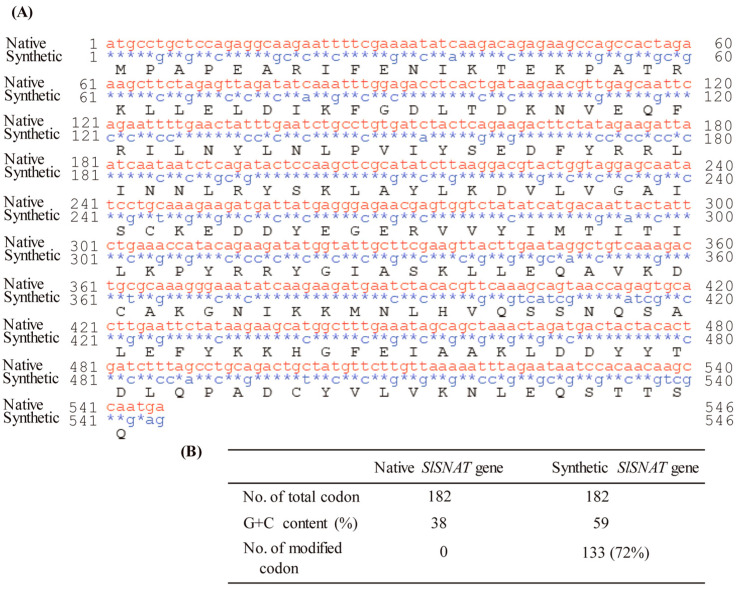
(**A**) Nucleotide alignment between native (red; CDW73552) and synthetic (blue) *S. lemnae SNAT*. Identity is denoted by stars. Black letters, amino acids. (**B**) Modification of *S. lemnae SNAT* codons. The nucleotide sequence of synthetic *S. lemnae SNAT* was manually codon optimized with reference to the rice *SNAT2* codon.

To verify its function, we first synthesized the full-length coding sequence (CDS) of the *SlSNAT* gene in accordance with rice *SNAT2* codon usage (GenBank accession number AK068156) for efficient *SlSNAT* gene expression in rice plants. The complete 546 nucleotides of the synthetic *SlSNAT* CDS are shown in Figure 2A. Changing the third codon position from A or T to G or C increased the G+C content of synthetic *SlSNAT*. Therefore, a total of 133 of 182 codons were modified. As expected, the G+C content of synthetic *SlSNAT* increased to 59% (Figure 2B), in contrast to 38% for native *SlSNAT*, and was therefore much closer to that of rice *SNAT2* (70%).

### 3.2. Purification of Recombinant SlSNAT and Enzyme Kinetic Analysis

**Figure 3:**
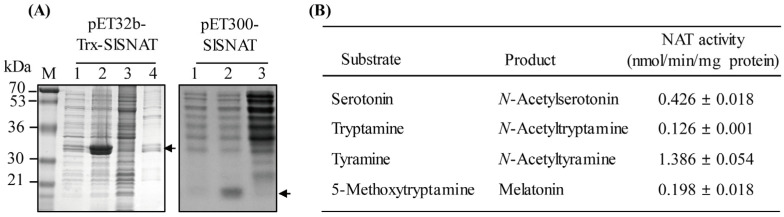
*Escherichia coli* expression, affinity purification of SlSNAT recombinant protein, and its enzymatic characteristics. (**A**) Expression of SlSNAT as a thioredoxin (Trx) fusion protein using a pET32b vector and expression of SlSNAT as an N-terminal His × 6-tagged SlSNAT protein using a pET300 vector. (**B**) Serotonin *N*-acetyltransferase enzyme activity (SNAT) as a function of various substrates. The expression of recombinant SlSNAT protein is marked by arrows.

The synthetic full-length *SlSNAT* CDS was first cloned into pET300 for expression with an N-terminal hexa-histidine tag and purified by Ni^2+^ affinity column chromatography. However, this recombinant SlSNAT protein was insoluble and could not be purified (Figure 3A). To enhance solubility, we used a thioredoxin (Trx)-tagged SlSNAT expression system employing the pET32b vector. The soluble Trx-SlSNAT recombinant protein was purified by Ni^2+^ affinity column chromatography, although the majority of the expressed protein remained insoluble (Figure 3A). The purified recombinant Trx-SlSNAT protein was first examined for SNAT enzyme activity in catalyzing the conversion of serotonin to NAS. As shown in Figure 3B, the recombinant SlSNAT exhibited SNAT-specific enzyme activity of 7.1 pkat/mg protein, which was similar to the activity reported previously for an archaeon SNAT (6.7 pkat/mg protein) [20]. The SNAT enzyme activity of SlSNAT was 1.8-fold higher than that of *E. coli* RimI [21] but 4.7-fold lower than that of rice SNAT3 [28]. All SNAT enzymes from animals or plants can accept many other amines as substrates [16,30]. The highest SNAT enzyme activity was observed with the tyramine substrate, followed in order by serotonin, 5-MT, and tryptamine (Figure 3B). Both archaeon SNAT and rice SNAT3 showed the highest SNAT enzyme activity toward tyramine as a substrate, whereas *E. coli* RimI showed preference for 5-MT over other amines. The biological significance of substrate preference among SNAT enzymes has not been elucidated. Based on these observations, SlSNAT was confirmed to exhibit SNAT enzyme activity. These results suggested that *S. lemnae* can directly synthesize melatonin in the presence of 5-MT and indirectly synthesize melatonin via NAS. Consistent with mechanisms observed in other organisms, *S. lemnae* can synthesize melatonin by two pathways: from serotonin to NAS and melatonin, and from serotonin to 5-MT and melatonin.

**Figure 4:**
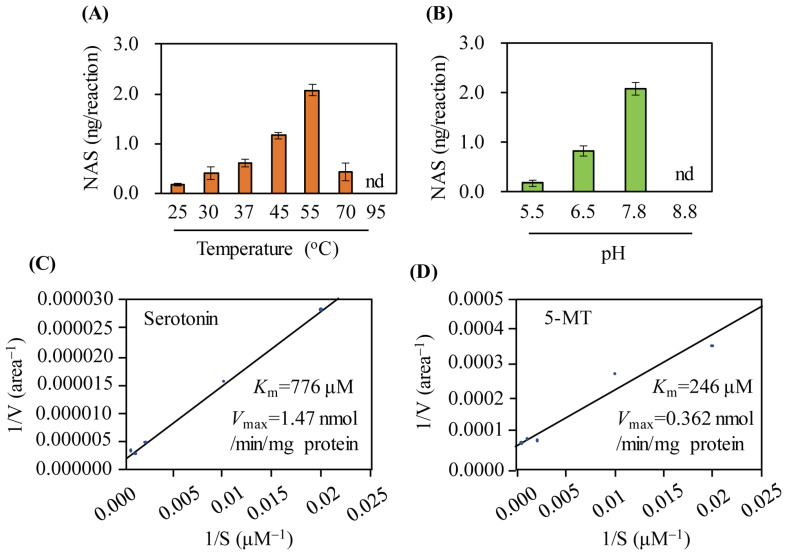
SNAT enzyme kinetic analysis. Serotonin *N*-acetyltransferase enzyme activity as a function of (**A**) various temperature, (**B**) various pH, (**C**) *K*_m_ and *V*_max_ values for serotonin substrate, (**D**) *K*_m_ and *V*_max_ values for 5-methoxytryptamine (5-MT) substrate. Values are means ± SD (n = 3). nd, not detectable.

Similar to other SNAT enzymes, such as plant SNAT, human Naa50, and *E. coli* RimI, the optimal temperature and pH of SlSNAT toward serotonin as a substrate were 55 °C and pH 7.8, respectively (Figure 4A,B) [16,18,21]. The *K*_m_ and *V*_max_ values of SlSNAT toward serotonin as a substrate were 776 µM and 1.47 nmol/min/mg protein, respectively (Figure 4C). For 5-MT as a substrate, SlSNAT exhibited *K*_m_ and *V*_max_ values of 246 µM and 0.362 nmol/min/mg protein, respectively (Figure 4D). The catalytic efficiency (*V*_max_/*K*_m_) was slightly higher toward serotonin than toward 5-MT, suggesting that SlSNAT shows substrate preference for serotonin over 5-MT during melatonin biosynthesis. However, in the presence of lower substrate concentrations, SlSNAT preferentially utilized 5-MT during melatonin synthesis because it showed higher substrate affinity for 5-MT than for serotonin. Further in-depth studies are required to determine whether SlSNAT exhibits protein NAT activity similar to that of human Naa50 [18].

### 3.3. Transgenic Rice Plants Overexpressing SlSNAT

**Figure 5:**
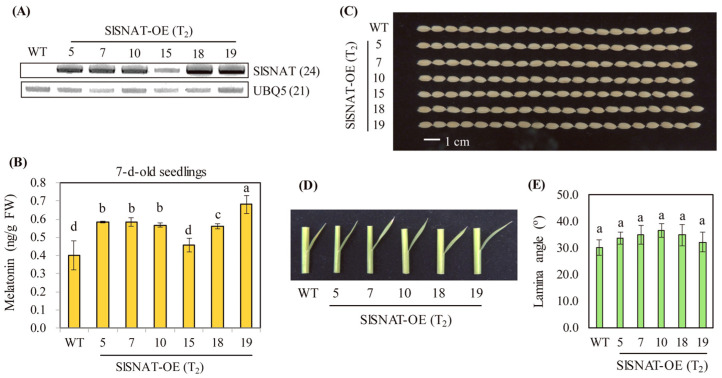
Generation of *SlSNAT* overexpression transgenic rice and the melatonin content of rice seedlings. (**A**) RT-PCR analyses of transgenic and wild-type 7-day-old rice seedlings. (**B**) Melatonin contents of 7-day-old rice seedlings. (**C**) Photograph of seed length. (**D**) Photograph of lamina angle in 3-week-old rice seedling. (**E**) Measurement of lamina angle. WT, wild type; *UBQ5*, rice ubiquitin 5 gene. GenBank accession number of *UBQ5* is AK061988. Different letters indicate significant differences among lines (Tukey’s HSD; *p* < 0.05).

To investigate the biological function of *SlSNAT*, ectopic overexpression of *SlSNAT* in rice was performed under the control of the constitutive maize ubiquitin promoter. A total of 19 independent transgenic lines were generated through *Agrobacterium*-mediated transformation. Six homozygous lines of T_2_ seeds were further selected to examine the gain-of-function effects of *SlSNAT* expression on melatonin synthesis in rice. To confirm the ectopic overexpression of the *SlSNAT* transgene mRNA, reverse transcription polymerase chain reaction (RT-PCR) analysis was performed in the *SlSNAT*-overexpressing (SlSNAT-OE) transgenic rice plants. *SlSNAT* mRNA was detected in total RNA isolated from the leaves of 7-day-old transgenic rice seedlings. All except one transgenic line (line 15) exhibited a high level of transgene expression, whereas no detectable *SlSNAT* mRNA was observed in wild-type controls (Figure 5A).

To determine whether *SlSNAT* overexpression was functionally associated with melatonin synthesis, the levels of melatonin were measured in 7-day-old seedlings of SlSNAT-OE lines. As shown in Figure 5B, all transgenic lines (with the exception of line 15) produced higher levels of melatonin than wild-type control, indicating a positive correlation between *SlSNAT* mRNA expression and melatonin level. It was previously reported that rice *SNAT2* downregulation resulted in short grains, in conjunction with a decrease in brassinosteroid (BR) level [31]. As a simple phenotypic test for increased BR level, grain length was first monitored in the SlSNAT-OE lines. As shown in Figure 5C, some transgenic lines (e.g., lines 7, 18, and 19) exhibited a slight increase in grain length, whereas other lines (e.g., lines 5, 10, and 15) showed similar grain length to the wild-type control. These results indicated that BR levels were not significantly increased in the SlSNAT-OE lines relative to the wild type. Lamina angle (the angle between the second leaf blade and vertical culm) was monitored as another BR indicator because BR plays a key role in lamina angle increase. As shown in Figure 5D,E, the lamina angles of the SlSNAT-OE lines were comparable to those of wild-type controls, indicating that BR levels were not elevated in the SlSNAT-OE lines; this finding was similar to previous results from transgenic rice plants overexpressing rice *SNAT2* [31]. Taken together, these observations indicated that an endogenous melatonin increase is not necessarily coupled to an increase in BR.

### 3.4. Elevated Melatonin Levels Confer Senescence Tolerance

Melatonin acts as a potent antioxidant that scavenges many oxidants, including reactive oxygen species (ROS) and reactive nitrogen species (RNS) [9]. Aging and senescence are major physiological consequences of a lack of antioxidant activity in both animals and plants [32,33]. To determine whether SlSNAT-OE transgenic rice plants exhibited senescence tolerance mediated by increased melatonin levels, detached rice leaves were subjected to senescence treatment.

**Figure 6:**
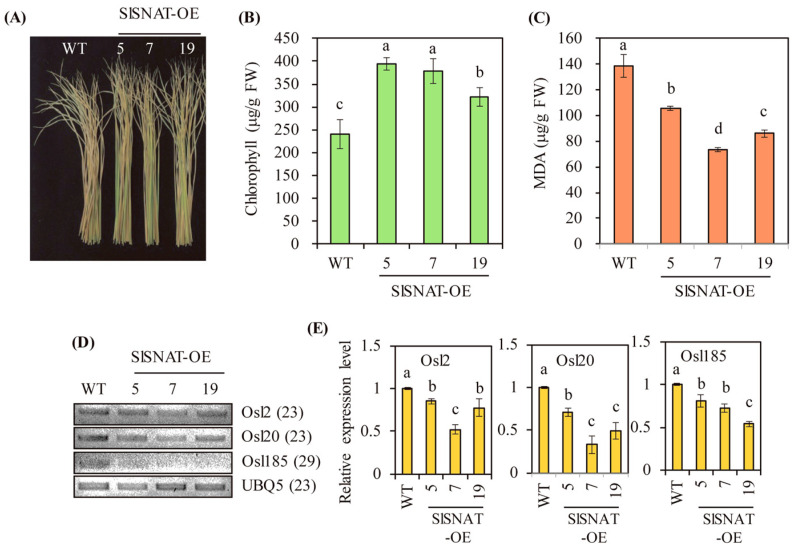
Enhanced senescence tolerance in *SlSNAT*-overexpressing transgenic rice plants. (**A)** Photograph of senescence-treated 5-week-old rice leaves. (**B**) Chlorophyll contents in senescence-treated rice leaves. (**C**) Malondialdehyde (MDA) contents. (**D**) Gene expression profiles of senescence marker genes by RT-PCR. (**E**) Gene expression profiles of senescence marker genes by quantitative RT-PCR. Fourth and fifth leaves from 5-week-old rice plants grown in soil pots were detached and this was followed by senescence treatment for 12 days. WT, wild type; *UBQ5*, rice ubiquitin 5 gene. GenBank accession numbers are *Osl2* (AF251073), *Osl20* (AF251067), *Osl185* (AF251075), and *UBQ5* (AK061988). Different letters indicate significant differences among the lines (Tukey’s HSD; *p* < 0.05).

As shown in Figure 6A, the SlSNAT-OE lines exhibited delayed leaf senescence, indicated by increased chlorophyll levels compared with wild-type control (Figure 6B). In parallel with the increased chlorophyll levels, the contents of malondialdehyde (MDA), one of the end products of lipid peroxidation, were decreased in the SlSNAT-OE lines relative to wild type, indicating that the SlSNAT-OE lines displayed oxidative stress tolerance (Figure 6C). Consistent with the biochemical data highlighting senescence tolerance, several senescence marker genes (e.g., *Osl2*, *Osl20*, and *Osl185* [34]) were expressed at lower levels in the SlSNAT-OE lines than in wild-type controls, as determined by RT-PCR and quantitative RT-PCR (qRT-PCR) (Figure 6D,E). Taken together, these data showed that the elevated endogenous melatonin levels mediated by *SlSNAT* overexpression in the transgenic rice plants conferred tolerance against senescence. This was mainly attributed to the enhanced synthesis of melatonin, a potent antioxidant that efficiently scavenges ROS.

### 3.5. Melatonin Confers Tolerance against the Peroxidizing Herbicide Butafenacil

**Figure 7:**
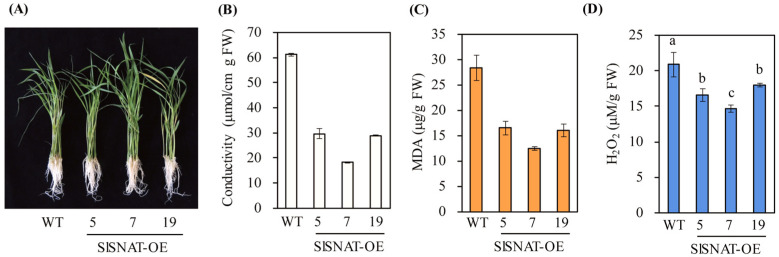
Enhanced tolerance of SlSNAT-overexpressing transgenic rice plants against peroxidizing herbicide butafenacil. (**A**) Photograph of rice seedlings after butafenacil treatment. (**B**) Effect of butafenacil treatment on cellular leakage. (**C**) MDA production from butafenacil-treated rice seedlings. (**D**) H_2_O_2_ content from butafenacil-treated rice seedlings. Seven-day-old rice seedlings were challenged with 0.1 µM butafenacil for 48 h. WT, wild type; FW, fresh weight. Different letters indicate significant differences among the lines (Tukey’s HSD; *p* < 0.05).

Butafenacil is a peroxidizing herbicide that targets protoporphyrinogen oxidase involved in chlorophyll biosynthesis, resulting in massive production of ROS followed by cell death [35]. Due to the potent antioxidant activity of melatonin, it was expected that SlSNAT-OE lines would exhibit tolerance against butafenacil. As shown in Figure 7, the SlSNAT-OE lines showed herbicide tolerance as indicated by reduced levels of cellular leakage, MDA, and H_2_O_2_ production compared with the wild-type controls. These observations suggested that enhanced endogenous melatonin production is closely associated with oxidative stress tolerance upon exposure to the peroxidizing herbicide butafenacil. Similar results were also observed in transgenic rice seedlings overexpressing sheep *SNAT* [36].

## 4. Discussion

Melatonin acts as a master regulator in plant growth and development by orchestrating the expression of a diverse array of genes involved in primary and secondary metabolism [20,37]. SNAT plays roles in the penultimate and final steps of melatonin biosynthesis, depending on the substrate [38].

**Figure 8:**
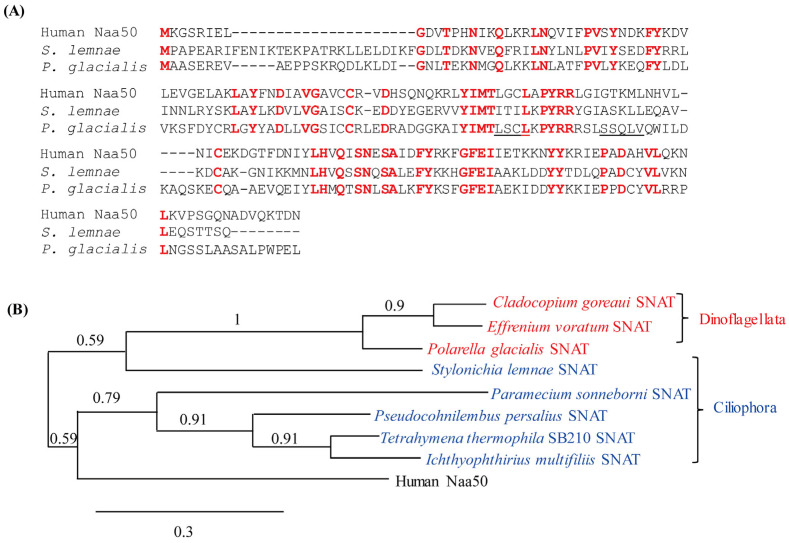
Sequence comparison and phylogenetic tree of SNAT in the Ciliophora and dinoflagellates. (**A**) Consensus amino acid sequences among three SNAT proteins including the human Naa50, the ciliate *Stylonichia lemnae* SNAT, and the dinoflagellate *Polarella glacialis* SNAT. Bold red letters indicate consensus amino acids. Dashes denote gaps for maximizing alignment of conserved residues. A coenzyme-A-binding pocket is underlined. (**B**) Phylogenetic tree analysis of SNAT proteins from the ciliates and dinoflagellates. GenBank accession numbers of various *SNAT* genes are as follows: human *Naa50* (BAB14397), *Cladocopium goreaui* (CAI3999280); *Effrenium voratum* (CAJ1361560); *Polarella glacialis* (CAK0876941); *Stylonichia lemna* (CCKQ01002460); *Paramecium sonneborni* (CAD8056267); *Pseudocohnilembus persalius* (KRX00195); *Tetrahymena thermophila* SB210 (XP_001025216); *Ichthyophthirius multifiliis* (XP-004035125). The scale bar represents 0.3 substitutions per site.

A novel clade of archaeal *SNAT* showing no sequence identity to either animal *AANAT* or plant *SNAT* genes was recently reported [20]. Thereafter, human *Naa50* as a functional orthologue of archaeal *SNAT* was confirmed to exhibit SNAT enzyme activity, and its ectopic overexpression was functionally linked to melatonin biosynthesis in rice [18]. Using human Naa50 as a query sequence, we screened for possible orthologues of *SNAT* genes in the alveolate subgroup of the Stramenopila, Alveolata, and Rhizaria (SAR) taxon. Alveolates comprise four major lineages: Chromerida, Apicomplexa, ciliates (Ciliophora), and dinoflagellates [1]. Many human *Naa50* orthologues were discovered in the genomes of alveolates with amino acid sequence identity ranging from 33% to 44%. Among them, the ciliate *S. lemnae* SNAT (SlSNAT) showed 38% identity to human Naa50, whereas the dinoflagellate *Polarella glacialis* SNAT had 36% identity. The predicted products of these SNAT orthologues showed high degrees of amino acid sequence identity although the coenzyme-A-binding pocket sequences were poorly conserved (Figure 8). On phylogenetic analysis, SlSNAT was closer to the dinoflagellate clade than the ciliate (Ciliophora) clade. Melatonin was first identified in the dinoflagellate *Lingulodinium polyedra* (also named *Gonyaulax polyedra*) in 1989 [39] and was subsequently found in many other dinoflagellate species, including *Symbiodinium* sp. [40,41]. Additionally, melatonin was quantified in the ciliate *Tetrahymena thermophila* [40]. Although melatonin was detected in these dinoflagellate and ciliate species, *SNAT* genes have not been cloned [1].

In the dinoflagellate *L. polyedra*, levels of both melatonin and 5-MT increased in response to low temperatures, in conjunction with the circadian rhythmicity of melatonin (showing a peak at night) [42,43]. Additionally, TPH, the first committed step enzyme for melatonin biosynthesis in animals, exhibited a circadian rhythm with high amplitude during the light period, antiphasic to the rhythm of melatonin [44]. The product of TPH enzyme catalysis is 5-hydroxytryptophan (5-HTP), which plays a key role in bioluminescence in *L. polyedra*. Furthermore, the dinoflagellate genus *Symbiodinium* exhibited melatonin rhythm with a nocturnal peak, although the diel pattern of melatonin levels did not persist under constant dark conditions [41]. The dark-induced melatonin increase is believed to be caused by the enhanced photoconsumption of melatonin by free radicals. Taken together, these observations indicated that melatonin can regulate circadian rhythm, as in *L. polyedra*; it also plays roles in antioxidant defense against free radicals generated from either cold stress or photosynthesis in these unicellular photosynthesizing algae [45].

By analogy, there is evidence that ciliate *T. thermophila* also produces melatonin, indicating that alveolates have a capacity to synthesize melatonin similar to the capacities of animals, plants, and fungi [10]. There have been no previous studies of melatonin biosynthesis in the ciliate *S. lemnae*, but the genome of *S. lemnae* reportedly carries an archaeal *SNAT* orthologue, the predicted product of which exhibits 38% amino acid identity to human Naa50. Purified recombinant SlSNAT protein has similar enzymatic characteristics (optimal pH, temperature, and substrate preference) to the archaeal SNAT orthologue protein products (Figure 3 and Figure 4), although there is some variation in kinetics among these proteins [18,20,21]. As for the possible function of melatonin in *S. lemnae*, it is presumed that melatonin may not only act as an antioxidant against various abiotic stresses but also be involved in protein quality control during growth as shown in plants and animals [7].

Eukaryotic phototrophs comprise three taxa: Excavata, SAR, and Archaeplastida. This is the first *SNAT* gene cloned in a eukaryotic phototroph outside the Archaeplastida. Melatonin synthesis and *SNAT* genes have been reported and cloned from two of the three Archaeplastida taxa: Rhodophyceae and Viridiplantae, but not Glaucophyta [11,46,47]. Melatonin was reported in the Excavata, such as *Euglena gracilis*, and in the SAR clade, including dinoflagellates and ciliates [1]. Because the dinoflagellates are ecologically important phytoplankton in marine environments and their genomes include *SNAT* orthologues (Figure 8), further detailed molecular genetic analyses based on these *SNAT* genes will provide new insights into the biological functions of melatonin in these organisms.

## 5. Conclusions

A novel archaeal *SNAT* clade was identified showing no apparent sequence identity to either animal *AANAT* or plant *SNAT*. Archaeal *SNAT* orthologues have recently been cloned from human [18], *E. coli* [21], and rice [28]. In this study, an orthologue of archaeal *SNAT* from *S. lemnae* was cloned, and its product was characterized. *SlSNAT* overexpression in the rice genome increased melatonin content relative to wild type. SlSNAT-OE rice plants exhibited increased tolerance to treatment with the peroxidizing herbicide butafenacil, as indicated by the lower levels of MDA and H_2_O_2_ compared with wild-type controls, indicating that as a potent antioxidant melatonin plays a role in defense against oxidative stress in rice by lowering ROS levels. However, we cannot rule out that other roles of melatonin, such as the induction of many antioxidant enzymes and protein quality control proteins, may also contribute to the beneficial effects of melatonin on oxidative stress.

## Data Availability

Data presented in this study are available within the article.
